# Knockdown of a Cyclic Nucleotide-Gated Ion Channel Impairs Locomotor Activity and Recovery From Hypoxia in Adult *Drosophila melanogaster*

**DOI:** 10.3389/fphys.2022.852919

**Published:** 2022-04-04

**Authors:** Shuang Qiu, Chengfeng Xiao, R. Meldrum Robertson

**Affiliations:** Department of Biology, Queen’s University, Kingston, ON, Canada

**Keywords:** insect, locomotion, hypoxia, ion channel, CNGL

## Abstract

Cyclic guanosine monophosphate (cGMP) modulates the speed of recovery from anoxia in adult *Drosophila* and mediates hypoxia-related behaviors in larvae. Cyclic nucleotide-gated channels (CNG) and cGMP-activated protein kinase (PKG) are two cGMP downstream targets. PKG is involved in behavioral tolerance to hypoxia and anoxia in adults, however little is known about a role for CNG channels. We used a CNGL (CNG-like) mutant with reduced *CNGL* transcripts to investigate the contribution of CNGL to the hypoxia response. CNGL mutants had reduced locomotor activity under normoxia. A shorter distance travelled in a standard locomotor assay was due to a slower walking speed and more frequent stops. In control flies, hypoxia immediately reduced path length per minute. Flies took 30–40 min in normoxia for >90% recovery of path length per minute from 15 min hypoxia. CNGL mutants had impaired recovery from hypoxia; 40 min for ∼10% recovery of walking speed. The effects of CNGL mutation on locomotor activity and recovery from hypoxia were recapitulated by pan-neuronal *CNGL* knockdown. Genetic manipulation to increase cGMP in the CNGL mutants increased locomotor activity under normoxia and eliminated the impairment of recovery from hypoxia. We conclude that CNGL channels and cGMP signaling are involved in the control of locomotor activity and the hypoxic response of adult *Drosophila*.

## Introduction

Insects have evolved remarkable adaptive mechanisms to tolerate hypoxia or anoxia. Apart from a few specialized, hypoxia-tolerant species, anoxia in vertebrates leads to irreversible tissue damage or death within minutes. However, the median lethal time (LT50) of midge, *Chironomus plumosis*, larvae exposed to anoxia can be as long as ∼200 days (Nagell and Landahl, 1978). Also, the larvae of tiger beetles, *Cicindela togata*, survive anoxia at 25°C with an LT50 of more than three days (Hoback et al., 1998; Hoback et al., 2000) and locusts, *Locusta migratoria,* are able to survive exposure to 100% nitrogen for up to six hours at room temperature (Wu et al., 2002). Similarly, adults of fruit flies, *Drosophila melanogaster,* can survive several hours in anoxia (Haddad, 2006; Callier et al., 2015; Campbell et al., 2018; Campbell et al., 2019b). At extremely low oxygen tension (0.1 kPa), flies reduce metabolism around 10-fold relative to normoxia (Van Voorhies, 2009). The exceptional survival and physiological responses to hypoxia or anoxia in *Drosophila* provide a good opportunity to explore its underlying molecular and genetic basis (Campbell et al., 2019a).

Rapid behavioral and electrophysiological responses to hypoxia involve the regulation of ion channels by reduced O_2_ levels (López-Barneo, 1996). The first identified ion channel responsible for O_2_ sensing was a voltage-dependent K^+^ channel (López-Barneo et al., 1988) found in Type I chemoreceptor cells in the mammalian carotid body. Hypoxia (*P*O_2_ dropping from 150 to 10 mm Hg) reduces the K^+^ current through the channel by 25–50%. Another O_2_-sensing channel is the O_2_-sensitive ATP-inhibitable K^+^ channel, which is found in neocortical and substantia nigra neurons in the rat CNS (Jiang et al., 1994; Jiang and Haddad, 1994). Hypoxia activates this channel and causes it to open and close with increased frequency. Moreover, in rat hippocampal CA1 neurons, the voltage-dependent, fast-inactivating Na^+^ inward current and neuronal excitability are depressed with decreased O_2_ levels (Cummins et al., 1991). In *Caenorhabditis elegans*, the hypoxia responses require cyclic guanosine monophosphate (cGMP)-gated cation channels (CNGs) including tax-2 and tax-4, and the atypical soluble guanylyl cyclases (sGCs) such as GCY-35 (Gray et al., 2004; Chang et al., 2006; Zimmer et al., 2009).

In *Drosophila*, oxygen sensing and behavioral responses to hypoxia were undescribed until a class of sGCs was identified, including Gyc88E, Gyc89Da and Gyc89Db (Morton, 2004) which contribute to the hypoxia response in *Drosophila* larvae (Vermehren-Schmaedick et al., 2010). *Drosophila* has a family of 4 different CNG channel genes, one of which, CNGA, regulates an escape response to hypoxia in *Drosophila* larvae (Vermehren-Schmaedick et al., 2010). However, similar responses have not been attributed to the other CNG channel family members, such as CNG-like (CNGL), CG3536 and CG17922. Flies with down-regulation of cGMP-specific phosphodiesterase (PDE) recover locomotor ability rapidly from anoxia, suggesting the involvement of cGMP in the modulation of anoxia recovery speed in adult flies (Xiao and Robertson, 2017). In *Drosophila* larvae, reduced levels of cGMP in O_2_-sensitive neurons result in longer times to respond to hypoxia, indicating that cGMP also regulates the escape response to hypoxia in *Drosophila* larvae (Vermehren-Schmaedick et al., 2010). cGMP has several downstream targets, such as CNG channels and cGMP-dependent protein kinase G (PKG). Flies with lower PKG activity show an increased time to the onset of anoxic coma and are more behaviorally resistant to anoxia and hypoxia (Dawson-Scully et al., 2010; Spong et al., 2016). However, whether CNG channels regulate the anoxia or hypoxia responses of adult flies is unknown. Under anoxia, neural activity is shutdown (Robertson et al., 2020), whereas under hypoxia, the CNS remains functional, indicating that it is feasible to investigate the role of CNG channels in maintaining neural function and regulating locomotor activity under hypoxia. To date, one of the CNG channel family members, the CNGL channel, has received little attention.

*CNGL* is detected in fly optic lobe, central brain and thoracic ganglia, shows similarity to the mammalian CNG channel *a* and *ß* subunits, and is predicted to form heteromeric channels with similar sensitivity to cAMP and cGMP (Miyazu et al., 2000; Vermehren-Schmaedick et al., 2010). The CNGL channel does not contribute to the hypoxia escape response in *Drosophila* larvae (Vermehren-Schmaedick et al., 2010). However, *CNGL* knockdown reduced visual orientation memory (Kuntz et al., 2017) and a *CNGL* homolog in Hawaiian crickets is a candidate gene underlying interspecific variation in centrally-generated song patterns (Xu and Shaw, 2019) indicating a role in motor patterning in the nervous system.

Considering that cGMP and PKG are involved in responses to hypoxia and that CNG channels contribute to the escape response to hypoxia in *Drosophila* larvae, yet CNGL channels do not, we investigated the role of CNGL channels in regulating the hypoxia response of adult flies. We hypothesized that mutation of *CNGL* would alter responses to hypoxia and tested the hypothesis using a locomotor assay and comparing *CNGL* mutant flies in a w1118 genetic background and w1118 control flies under normoxia, under hypoxia, and during recovery. In addition, we examined the effects of hypoxia on locomotor activity in pan-neural or pan-glial *CNGL* knockdown flies. Finally, we used fly lines with overexpression of Gyc88E (soluble guanylate cyclase) or mutation of a phosophodiesterase, *Pde1c,* to examine the possible interaction between CNGL and cGMP in response to hypoxia.

## Materials and Methods

### Flies

Fly strains and their sources: w1118 (L. Seroude laboratory, Queen’s University, Canada); w1118 Mi{ET1}CNGL^MB01092^ (Bloomington Stock Center, BSC #22988; culled from their stocks in 2017); UAS-CNGL-RNAi (BSC #28684); UAS-Gyc88E (D. Morton laboratory, Oregon Health & Science University, United States); Pde1c^KG05572^ (BSC #13901); elav-Gal4 (BSC #8765); and repo-Gal4 (BSC #7415).

Male progeny (CNGL^MB01092^/y) were obtained by crossing female CNGL^MB01092^ flies with male w1118 flies. This was to reduce as much as possible any differences in the genetic background of CNGL^MB01092^ and w1118 flies. Male progeny (CNGL^MB01092^/y; Pde1c^KG05572^/+) were obtained by crossing female CNGL^MB01092^ flies with male Pde1c^KG05572^ flies. These Pde1c^KG05572^ flies were confirmed in previous investigations in our laboratory to have reduced Pde1c transcript levels (Xiao and Robertson, 2017). Further synchronization of the genetic background was difficult due to the lack of an independent eye-color marker in the CNGL^MB01092^.

Up- or down-regulation of a gene was carried out using the Gal4/UAS binary expression system (Brand and Perrimon, 1993; Clemens et al., 2000; Duffy, 2002; Hammond et al., 2000). Male progeny (;elav-Gal4/+; UAS-CNGL-RNAi/+ and ;repo-Gal4/+; UAS-CNGL-RNAi/+) were obtained by crossing female UAS-CNGL-RNAi flies with elav-Gal4 or repo-Gal4 male flies, respectively. CNGL^MB01092^ mutants were originally generated by transgenic insertion of a *Minos* enhancer trap construct encoding a Gal4 driver (Metaxakis et al., 2005; Bellen et al., 2011). Therefore, to upregulate Gyc88E, male progeny (CNGL^MB01092^/y; UAS-Gyc88E/+) were obtained by crossing female CNGL^MB01092^ flies with UAS-Gyc88E male flies.

Flies were raised on standard medium (0.01% molasses, 8.2% cornmeal, 3.4% killed yeast, 0.94% agar, 0.18% benzoic acid, 0.66% propionic acid) at room temperature 21–23°C, 60–70% humidity. A 12h/12 h light/dark cycle was provided by three light bulbs (Philips 13 W compact fluorescent energy saver) with lights on at 7 am and off at 7 pm. Male flies were collected within two days after eclosion and raised for at least three additional days before experiments. 100% N_2_ gas was used to knock down flies during collection but flies were not exposed to 100% N_2_ for 3 days prior to testing. All experiments took place between 10 am and 4 pm on flies younger than 9 days old.

### RNA Extraction and RT-PCR

Total RNA from around 20 whole flies of both sexes at 4–7 days old was extracted using a PureLink RNA Mini Kit (Cat# 12183020, ThermoFisher Scientific, United States). Reverse transcription (RT) was performed using a GoScriptTM Reverse Transcription System (A5001, Promega, United States). Polymerase chain reaction (PCR) conditions were: 95°C for 30 min for hot start, followed by denaturing at 95°C for 30 s, annealing at 60°C for 1 min, extension at 72°C for 2 min for 40 cycles, and a final extension at 72°C for 10 min. The primers were: CNGL-RF (5′-TCG​TTC​ATC​AGC​GAG​CAT​CC-3′, 5′-GGT​GGC​AAC​GTT​CCT​CTT​GA-3′); CNGL-RD, RE, RJ and RI (5′-GGA​GAG​CTT​CGC​GTT​TCC​TG-3′, 5′-GAG​GAT​GAG​GAT​GTC​GGT​GC-3′). Tubulin 84B was used as loading control with primers 5′-CAT​GGT​CGA​CAA​CGA​GGC​TA-3′ and 5′-GCA​CCT​TGG​CCA​AAT​CAC​CT-3′. PCR products were separated in 1–2% agarose gel, stained with 0.5 μg/ml ethidium bromide and visualized with UV light. Images of DNA bands were captured with AlphaImager Image Analysis System (Alpha Innotech).

### Locomotor Assay

The locomotor assay has been described (Xiao and Robertson, 2015). Illumination for video-capture (frame rate of 15 frames/s) was provided with a white light box (Logan Portaview slide/transparency viewer with 15-W color-corrected fluorescent bulbs) and the reflection from white cardboard screens surrounding the set up. Individual flies were gently aspirated into circular arenas (1.27 cm diameter; 0.3 cm depth) that prevented flying but allowed their walking to be video recorded for later analysis. A custom-written fly tracking software using Open Computer Vision 2.0 (OpenCV2.0) was used for computing fly positions (the center of mass) and calculating path length per minute. Gas (air or the hypoxia mixture) continuously flowed through the apparatus at 2 L/min. After 5 min of adaptation in the arena and 10 min of locomotor activity recorded under normoxia, hypoxia was induced by switching from air to a mixture of 2% O_2_ and 98% N_2_ for 15 min. These parameters for the hypoxia were chosen based on preliminary results indicating a good balance between the severity of the effect and the ability to recover in a short enough time to prevent the flies being stressed (dehydration and starvation) in the locomotor chambers. The ratio of O_2_ and N_2_ chosen here was based on previous studies, which define such a composition as “hypoxia” (Allweis et al., 1984; Donohoe et al., 2000). After switching back to air, the flies recovered under normoxia for 40 min. Experiments were conducted in the light and at least three hours away from light-dark transit, in order to avoid morning and evening activity peaks (Grima et al., 2004).

Every video recording was inspected to ensure that the flies were walking apparently normally in the arenas throughout the experiment and not changing position due to uncoordinated movements. This pertains particularly to repo-Gal4/UAS-CNGL-RNAi flies in which locomotor activity was severely impaired and they were excluded from the analysis of recovery from hypoxia. For this genotype, the large transient changes in path length represented movements associated with seizures and were evident only for the flies with *CNGL* knockdown in glia. Flies do not walk continuously in one direction in the arena but pause for variable periods and/or change direction (Xiao et al., 2018). To estimate the frequency of pausing during the first 10 min in normoxia, we counted the number of 1 s intervals during which the path length was zero (e.g. a fly motionless for 10 min would have 600 s of zero path length, represented as 600 stops/10 min). To estimate maximum walking speed, we chose the farthest distance travelled in 1 s during the first 10 min, assuming that during normoxia each fly would have at least one 1 s episode of continuous walking. Every minute the total path length, including stationary periods, was averaged and plotted under three different conditions including normoxia (10 min), hypoxia (15 min), and recovery after hypoxia (40 min). In addition, the path length per minute under normoxia was averaged over 10 min (1–10 min) (L_normoxia_), under hypoxia during the last 10 min (16–25 min) (L_hypoxia)_ and under normoxia during the first 10 min after 5 min adaptation (31–40 min) and the last 10 min of recovery (56–65 min) (L_recovery_). L_normoxia_ was compared within and between fly genotypes. To determine the effect of O_2_ level changes on fly locomotion, L_normoxia_, L_hypoxia_ and L_recovery_ were compared within the same fly line. Percent recovery was calculated only for the fly lines with significant effects of O_2_ level changes on fly locomotor activity (*p* < 0.05 when performing Kruskal–Wallis test). For each fly, the percent recovery after hypoxia was compared within and between fly genotypes using the values for L_normoxia_ and L_recovery_ (i.e. the final 10 min of recovery). At the end of the experiment, flies were removed from the locomotor assay arenas and discarded without further observation.

### Statistics

Three replicates of each experiment with groups of flies were conducted. The sample sizes for each test are stated in the Results section. It should be noted that, due to the inconsistent yields of progeny obtained after fly crosses, the sample sizes for the experiments varied. Statistical analysis was performed using Prism version 5.0 (GraphPad Software, San Diego, CA). A D’Agostino & Pearson omnibus normality test was conducted to examine the data distribution. Because some of the data had non-Gaussian distributions, nonparametric Mann-Whitney or Kruskal–Wallis tests with post-hoc comparisons were performed to examine the difference of medians between groups. Kruskal–Wallis test (without post-hoc comparisons) was performed to determine the effect of O_2_ level changes within the same fly line. *p* < 0.05 was considered as indicating statistical significance. Path lengths over time for different genotypes are displayed as mean ± standard error. Average path lengths during different stages of the experiment are displayed as medians with whiskers outlining the interquartile range, overlaid with individual data points.

## Results

### *CNGL* Transcriptional Down-Regulation in CNGL^MB01092^ Flies

**FIGURE 1:**
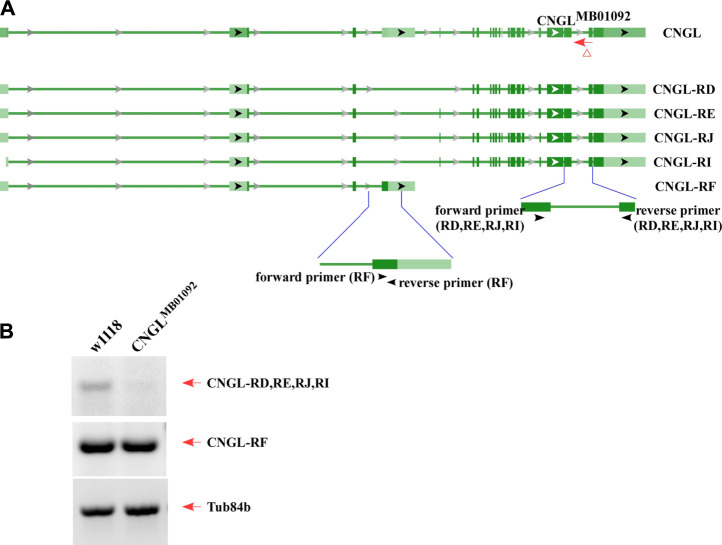
Low level of *CNGL* transcripts in CNGL^MB01092^. **(A)** Five predicted transcripts of *CNGL* (CNGL-RD, RE, RJ, RI and RF), two sets of primers and CNGL^MB01092^ mutant line. **(B)** RT-PCR was performed on total RNA extracted from whole flies of either w1118 or CNGL^MB01092^. For a loading control, the cDNA was also amplified with Tub84b primers. Lower level of *CNGL* transcripts was observed in the mutant.

RT-PCR was used to examine transcriptional changes in *CNGL* mutants. According to the National Center for Biotechnology Information (NCBI) nucleotide database, there are five mRNA variants including CNGL-RD, RE, RJ, RI and RF for *CNGL* (Figure 1A). Among them, CNGL^MB01092^ showed a reduction of CNGL-RD, RE, RJ and RI transcripts compared with w1118 (Figure 1B). The other variant, CNGL-RF, was likely unchanged. Thus, CNGL^MB01092^ flies had reduced levels of *CNGL* transcripts.

### Reduced Locomotor Activity Under Normoxia and Reduced Recovery From Hypoxia in CNGL^MB01092^/y Flies

**FIGURE 2:**
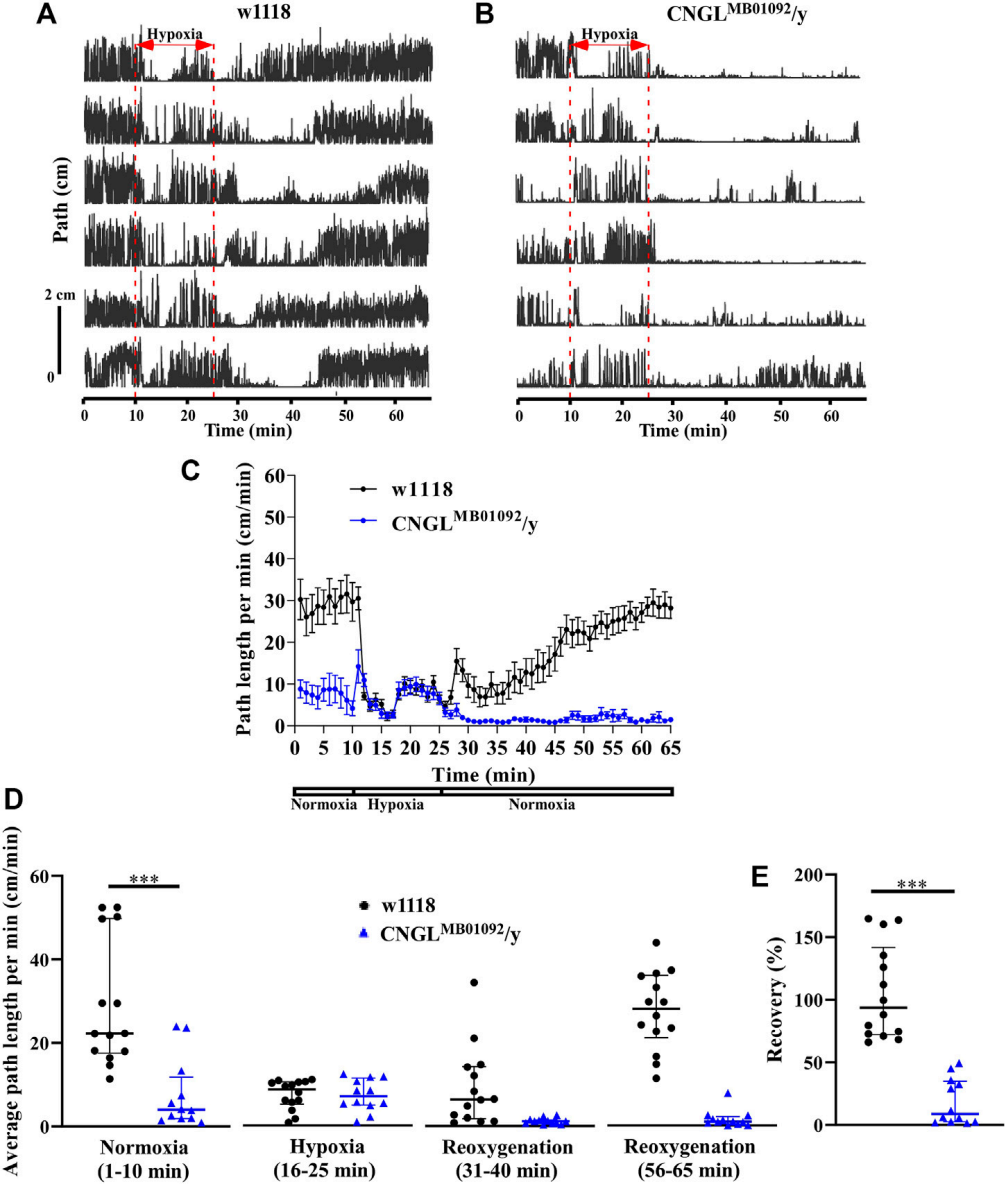
Reduced locomotor activity under normoxia and reduced recovery in response to hypoxia in CNGL^MB01092^/y flies. **(A,B)** Locomotor analysis for w1118 flies and CNGL^MB01092^/y flies with hypoxia. In this and following figures, each trace represents locomotor activity of a single fly illustrated by plotting the distance the fly’s centre of mass moved in successive 0.2 s intervals (Path) against the time of occurrence. A 15 min hypoxia was applied after 10 min locomotor activity under normoxia. Flies were then returned to normoxia and allowed to recover for 40 min. **(C)** Path length per minute of control flies (w1118) (black circles and black line, *n* = 14) and mutant fly line CNGL^MB01092^/y (blue circles and blue line, *n* = 12) under normoxia, during hypoxia and after hypoxia-treatment. **(D)** The average path length per minute under normoxia (1–10 min), during hypoxia (16–25 min), and during reoxygenation (31–40 min and 56–65 min) of w1118 and CNGL^MB01092^/y flies. **(E)** The percent recovery of w1118 and CNGL^MB01092^/y flies in response to hypoxia. Asterisks (***) indicate *p* < 0.001 by Mann-Whitney test.

Locomotor activity was analyzed under normoxia, under hypoxia, and during recovery (e.g., Supplementary Videos S1, S2, S3). During the 10 min period of normoxia, w1118 flies walked farther in each minute than CNGL^MB01092^/y flies (Figures 2A–C). Additionally, w1118 flies had significantly fewer stops of at least 1 s (*n* = 14, median 83 stops/10 min, interquartile range (IQR) 25.3-108.8 stops/10 min) than CNGL^MB01092^/y flies (*n* = 12, median 259.5 stops/10 min, IQR 170.3-368.5 stops/10 min) (*p* < 0.001, Mann-Whitney test). This measure does not discriminate between many short stops and few long stops. However, examination of the raw traces of path against (Figures 2A,B) shows no long periods of standing still. The maximum speed of the w1118 flies was also faster (maximum speed: *n* = 14, median 1.3 cm/s, IQR 1.2-1.5 cm/s) compared to CNGL^MB01092^/y flies (maximum speed: *n* = 12, median 0.8 cm/s, IQR 0.6-1.1 cm/s) (*p* < 0.001, Mann-Whitney test). Under hypoxia, the path length per minute of w1118 flies dropped, however, in CNGL^MB01092^/y flies it showed an acute increase within 1 min before dropping, which was followed by a gradual increase (Figures 2A–C). During the recovery period, *CNGL* mutants showed minimal activity, while w1118 flies had a larger path length per minute (Figures 2A–C).

Under normoxia, w1118 flies walked farther (*n* = 14, median 22.3 cm/min, IQR 17.6-49.8 cm/min) than CNGL^MB01092^/y flies (*n* = 12, median 4.0 cm/min, IQR 1.9-11.8 cm/min) (*p* < 0.001, Mann-Whitney test) (Figure 2D, left panel). Therefore, the *CNGL* mutation obtained from CNGL^MB01092^/y flies was associated with reduced locomotor activity under normoxia. Alterations of O_2_ level had a significant influence on fly locomotor activity in w1118 flies (*p* < 0.001, Kruskal–Wallis test without post-hoc test) (Figure 2D). Similarly, locomotor activity in CNGL^MB01092^/y flies also varied with the O_2_ level changes (*p* < 0.001, Kruskal–Wallis test) (Figure 2D). CNGL^MB01092^/y flies showed a reduced recovery (*n* = 12, median 8.9%, IQR 2.3-38.1%) compared with w1118 control flies (*n* = 14, median 93.9%, IQR 72.3-141.7%) (*p* < 0.001, Mann-Whitney test) (Figure 2E). Thus, the mutation in CNGL^MB01092^/y flies slowed locomotor activity under normoxia and led to reduced recovery from hypoxia.

### Reduced Locomotor Activity Under Normoxia and Reduced Recovery in Response to Hypoxia in Flies With *CNGL* Knockdown in Neurons

To examine the relationship between reduced locomotor activity under normoxia and fly genotypes, we first targeted RNAi knockdown of *CNGL* to the central nervous system with the pan-neuronal driver elav-Gal4 (Luo et al., 1994; Armstrong et al., 2011; Dimitroff et al., 2012). No apparent morphological abnormality or developmental delay was observed in the *CNGL* pan-neuronal knockdown flies compared with controls.

**FIGURE 3:**
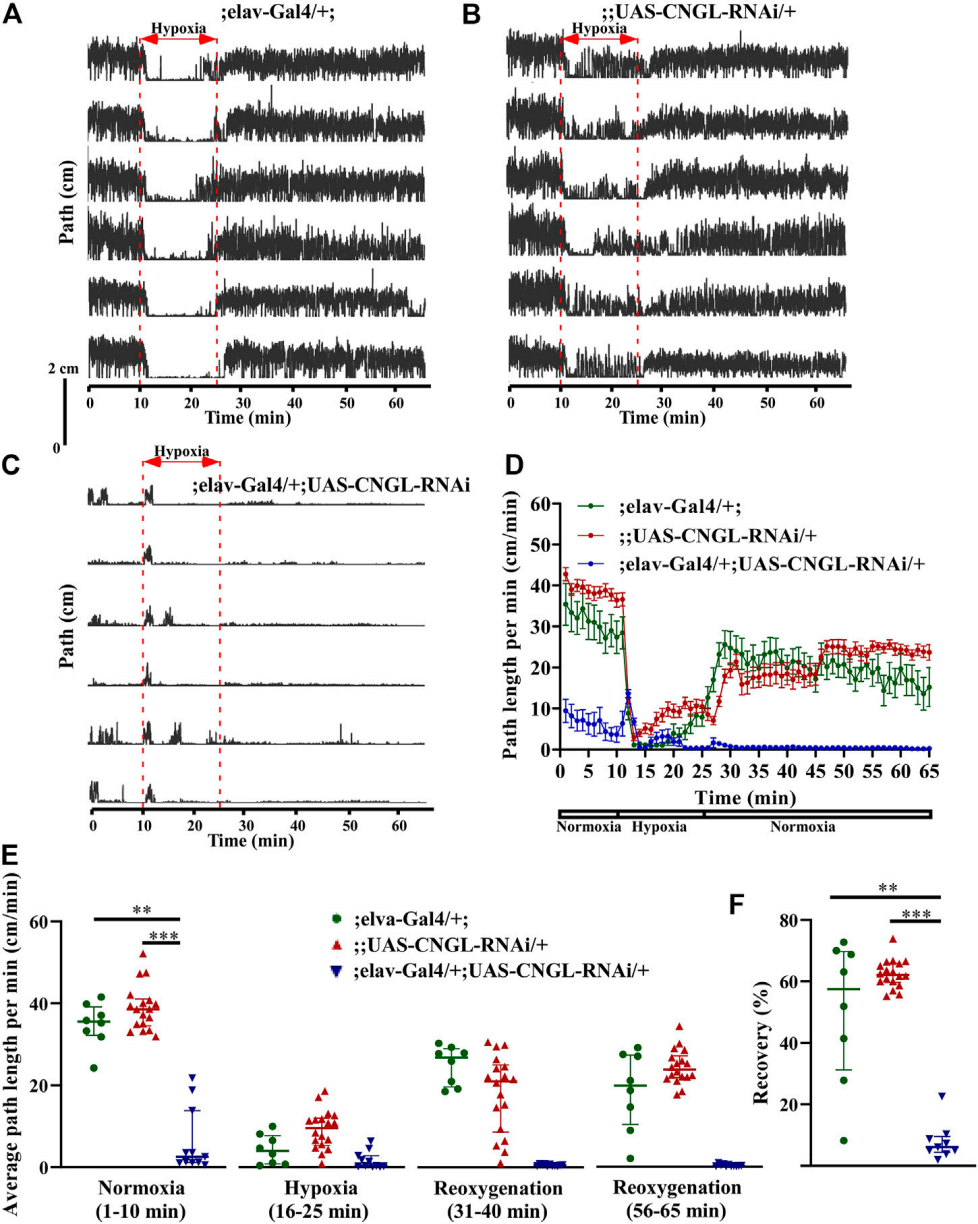
Reduced locomotor activity under normoxia and reduced recovery in response to hypoxia in flies with *CNGL* knockdown in neurons. **(A–C)** Locomotor analysis for flies with *CNGL* knockdown pan-neuronally and control flies with hypoxia. **(D)** Path length per minute of control flies (;elav-Gal4/+; , green circles and green line, *n* = 8, and ;UAS-CNGL-RNAi/+, red circles and red line, *n* = 18) and *CNGL* pan-neuronal knockdown flies (;elav-Gal4/+; UAS-CNGL-RNAi/+, blue circles and blue line, *n* = 11) during normoxia, hypoxia and recovery process. **(E)** The average path length per minute under normoxia (1–10 min), during hypoxia (16–25 min), and during reoxygenation (31–40 min and 56–65 min) of control flies and flies with *CNGL* knockdown in neurons. **(F)** The percent recovery of control and *CNGL* pan-neuronal knockdown flies. Asterisks (** or ***) indicate *p* < 0.01 or 0.001, respectively, by Kruskal–Wallis test with Dunn’s multiple comparison.

Under normoxia, the locomotor activity of ;elav-Gal4/+; UAS-CNGL-RNAi/+ males was less than controls (Figures 3A–D, Supplementary Video S4). The average path length per minute of the mutant flies (*n* = 11, median 2.6 cm/min, IQR 1.2-13.8 cm/min) was severely reduced compared with controls (;elav-Gal4/+; *n* = 8, median 35.5 cm/min, IQR 32.2-39.2 cm/min, and ;UAS-CNGL-RNAi/+, *n* = 18, median 38.6 cm/min, IQR 34.5-41.1 cm/min) (*p* < 0.01 or 0.001, respectively, Kruskal–Wallis test with Dunn’s multiple comparison) (Figure 3E, left panel). Like the CNGL^MB01092^/y flies, flies with *CNGL* knockdown in neurons also showed an acute increase in path length per minute at the beginning of hypoxia (Figures 3C,D). The O_2_ level changes had a significant effect on fly locomotor activities in all the three fly genotypes (*p* < 0.001 for all three comparisons within same genotype, Kruskal–Wallis test without post-hoc test) (Figure 3E). During the recovery after hypoxia, ;elav-Gal4/+; UAS-CNGL-RNAi/+ males showed reduced locomotor activity compared with controls (Figures 3A–D). The percent recovery of mutant flies (*n* = 11, median 6.1%, IQR 4.4-9.6%) was significantly lower than controls (;elav-Gal4/+;, *n* = 8, median 57.5%, IQR 31.3-69.8%, and ;UAS-CNGL-RNAi/+, *n* = 18, median 62.1%, IQR 59.7-65.9%) (*p* < 0.01 or 0.001, respectively, Kruskal–Wallis test with Dunn’s multiple comparison) (Figure 3F).

### Minimal Locomotor Activity Under Normoxia in Flies With *CNGL* Knockdown in Glia

**FIGURE 4:**
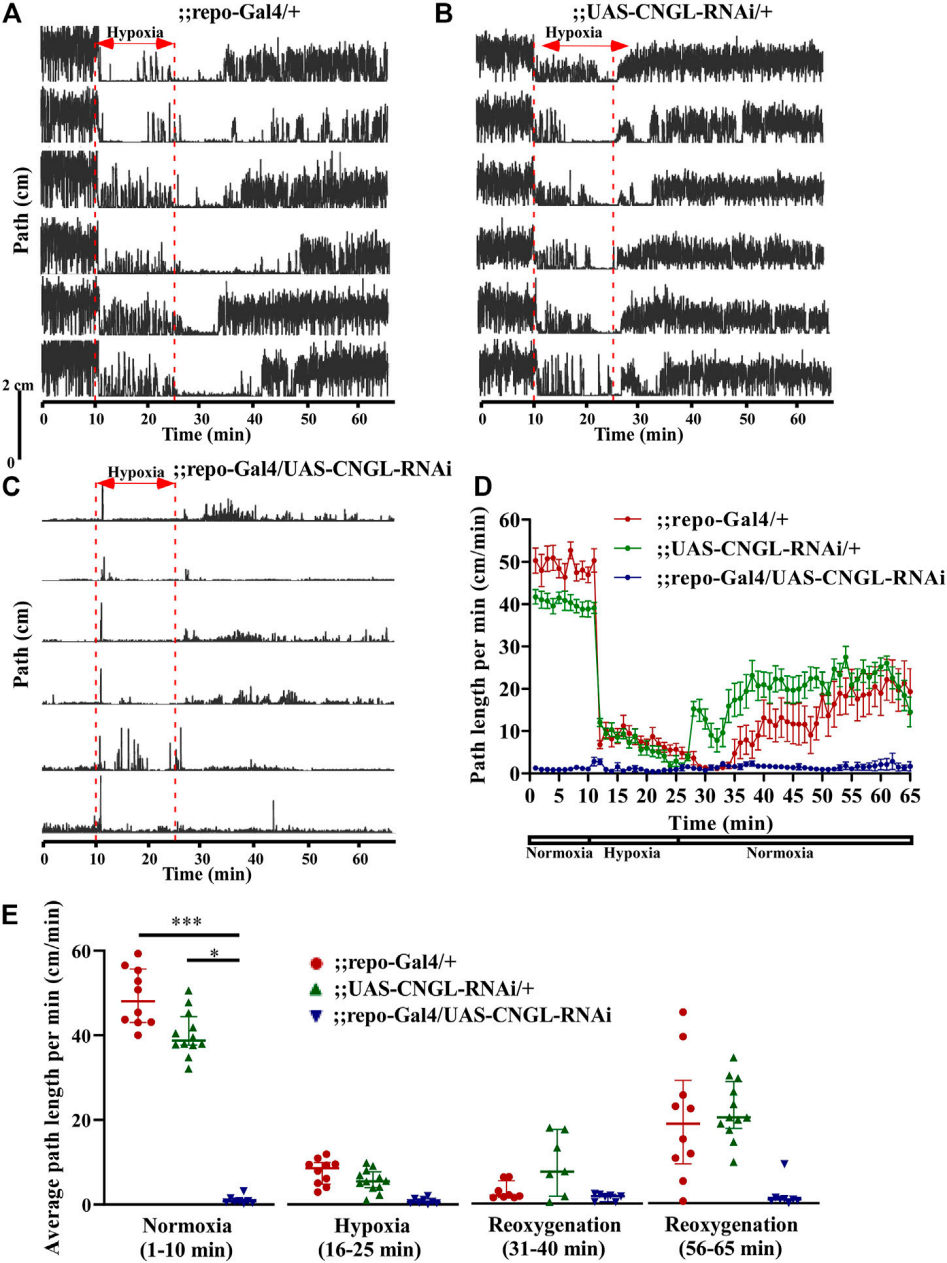
Reduced locomotor activity under normoxia in flies with *CNGL* knockdown in glia. **(A–C)** Locomotor analysis for flies with pan-glial *CNGL* down-regulation and control flies with hypoxia. **(D)** Path length per minute of control flies (;repo-Gal4/+; , red circles and red line, *n* = 10, and ;UAS-CNGL-RNAi/+, green circles and green line, *n* = 12) and *CNGL* pan-glial knockdown flies (;repo-Gal4/UAS-CNGL-RNAi, blue circles and blue line, *n* = 8) during normoxia, hypoxia and recovery process. **(E)** The average path length per minute under normoxia (1–10 min), during hypoxia (16–25 min), and during reoxygenation (31–40 min and 56–65 min) of control flies and flies with *CNGL* knockdown in glia. Asterisks (* or ***) indicate *p* < 0.05 or 0.001, respectively, by Kruskal–Wallis test with Dunn’s multiple comparison.

RNAi knockdown of *CNGL* using the pan-glial driver repo-Gal4 (Sepp et al., 2001) showed no apparent morphological abnormality or developmental delay. However, it led to greatly reduced locomotor activity compared with controls under normoxia (Figures 4A–D, Supplementary Video S5). ;;repo-Gal4/UAS-CNGL-RNAi males displayed significantly lower average path length per minute (*n* = 8, median 0.8 cm/min, IQR 0.4-1.3 cm/min) compared with controls (;repo-Gal4/+, *n* = 10, median 48.1 cm/min, IQR 43.1-55.7 cm/min, and ;UAS-CNGL-RNAi/+, *n* = 12, median 38.8 cm/min, IQR 37.7-44.4 cm/min) (*p* < 0.001 or 0.05, respectively, Kruskal–Wallis test with Dunn’s multiple comparison) (Figure 4E, left panel). The changes in O_2_ level affected locomotor activity only in the two control flies (;repo-Gal4/+ and ;UAS-CNGL-RNAi/+, both *p* < 0.001, Kruskal–Wallis test without post-hoc test), however, the minimal locomotor activity in ;repo-Gal4/UAS-CNGL-RNAi flies was unaffected when O_2_ level was altered (*p* > 0.05, Kruskal–Wallis test without post-hoc test) (Figure 4E). An acute increase in path length per minute representing seizures could be observed in ;;repo-Gal4/UAS-CNGL-RNAi flies at the beginning of hypoxia treatment (Supplementary Video S5; Figure 4C).

### Overexpression of Gyc88E Eliminated the Reduced Recovery in Response to Hypoxia in *CNGL* Mutant

cGMP regulates the behavioral responses to hypoxia in *Drosophila* larvae (Vermehren-Schmaedick et al., 2010), and modulates the onset of anoxic coma (Dawson-Scully et al., 2010) and the speed of anoxic recovery in adult flies (Xiao and Robertson, 2017). However, it is unclear whether cGMP modulates locomotor activity under normoxia and whether it regulates the hypoxia response in adult flies. Moreover, it would be possible for increased activation of a reduced number of channels in the mutant to mitigate the locomotor impairment. Therefore, an atypical soluble guanylyl cyclase Gyc88E, which catalyzes cGMP production, was expressed in *CNGL* mutant flies to examine whether cGMP upregulation could compensate for the effects of *CNGL* downregulation.

**FIGURE 5:**
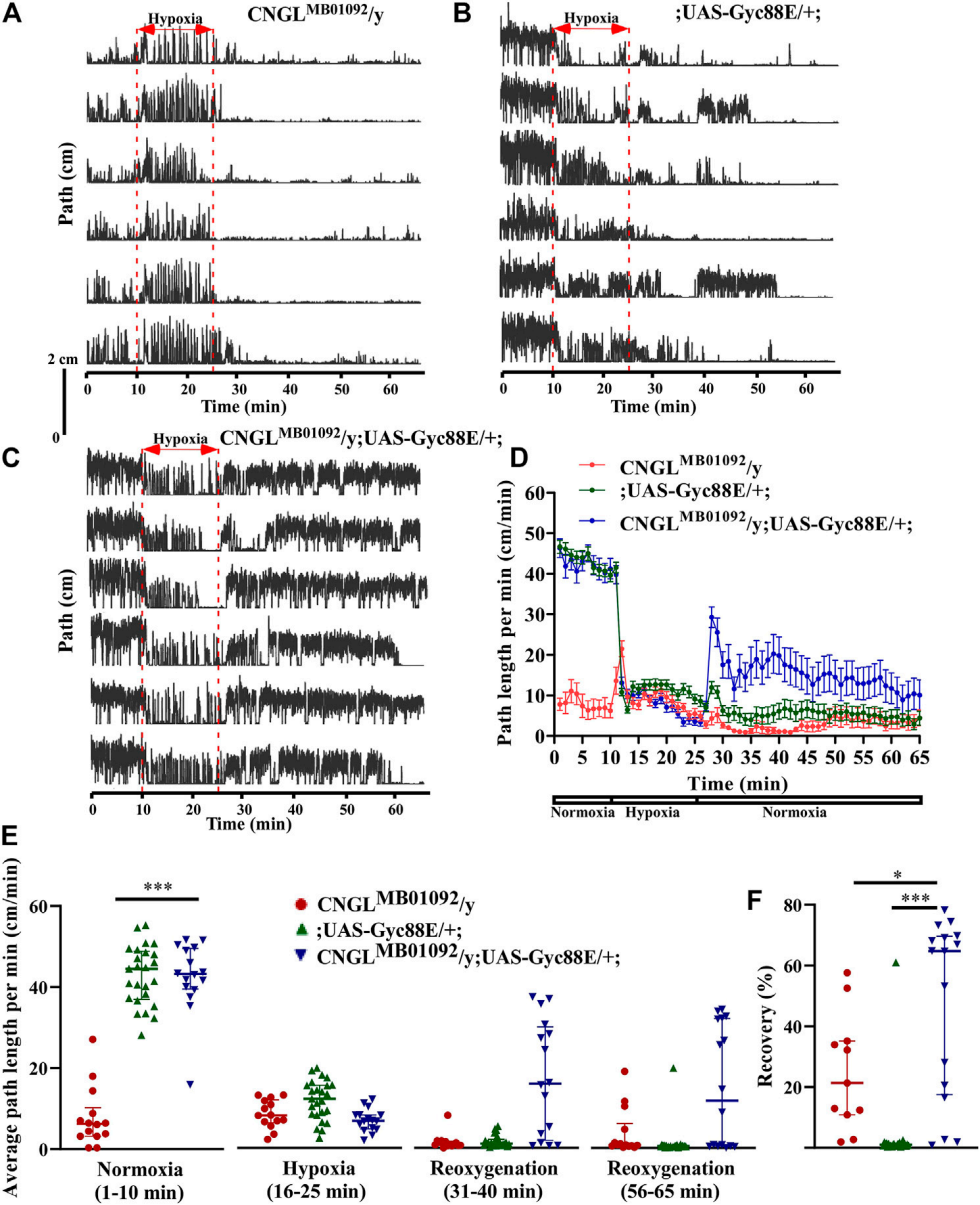
Overexpression of Gyc88E eliminated both the reduced locomotor activity under normoxia and the reduced recovery in response to hypoxia in CNGL mutant. **(A–C)** Locomotor analysis for flies with Gyc88E overexpression and control flies with hypoxia. **(D)** Path length per minute of control flies (CNGL^MB01092^/y, red circles and red line, *n* = 12, and ;UAS-Gyc88E/+; , green circles and green line, *n* = 29) and *CNGL* mutant flies with Gyc88E overexpression (CNGL^MB01092^/y; UAS-Gyc88E/+; , blue circles and blue line, *n* = 16) during normoxia, hypoxia and recovery. **(B)** The average path length per minute under normoxia (1–10 min), during hypoxia (16–25 min), and during reoxygenation (31–40 min and 56–65 min) of control flies and *CNGL* mutant flies with Gyc88E overexpression. **(C)** The percent recovery of control flies and *CNGL* mutant flies with Gyc88E overexpression. **(E)** The average path length per minute under normoxia (1–10 min), during hypoxia (16–25 min), and during reoxygenation (31–40 min and 56–65 min) of control flies and *CNGL* mutant flies with Gyc88E overexpression. **(F)** The percent recovery of control and *CNGL* mutant flies with Gyc88E overexpression. Asterisks (* or ***) indicate *p* < 0.05 or 0.001, respectively, by Kruskal–Wallis test with Dunn’s multiple comparison.

Under normoxia, the flies CNGL^MB01092^/y; UAS-Gyc88E/+; had improved locomotor activity compared with CNGL^MB01092^/y flies (Figures 5A–D). Although the average path length per minute of flies CNGL^MB01092^/y; UAS-Gyc88E/+; (*n* = 16, median 43.2 cm/min, IQR 39.5-49.5 cm/min) was comparable to flies ;UAS-Gyc88E/+; (*n* = 25, median 44.5 cm/min, IQR 36.9-48.9 cm/min) (*p* > 0.05, Kruskal–Wallis test with Dunn’s multiple comparison), the average speed of flies CNGL^MB01092^/y; UAS-Gyc88E/+; was significantly higher compared with CNGL^MB01092^/y flies (*n* = 14, median 6.2 cm/min, IQR 3.2-10.2 cm/min) (*p* < 0.001, Kruskal–Wallis test with Dunn’s multiple comparison) (Figure 5E, left panel). The alterations in O_2_ level affected locomotor activity in all three fly lines (*p* < 0.001 for all three comparisons within same genotype, Kruskal–Wallis test without post-hoc test) (Figure 5E). The CNGL^MB01092^/y flies also showed an acute increase in path length per minute within 2 min at the onset of hypoxia (Figures 5A,D).

During recovery, CNGL^MB01092^/y; UAS-Gyc88E/+; flies walked farther compared with the two control fly lines (Figures 5A–D). Overexpression of Gyc88E in *CNGL* mutant resulted in larger percent recovery (*n* = 16, median 64.7%, IQR 20.6-69.8%) while the two control fly lines, CNGL^MB01092^/y and ;UAS-Gyc88E/+;, had percentage recoveries of 21.3% (*n* = 14, IQR 6.9-43.8%), and 1.0% (*n* = 25, IQR 0.7-1.4%), respectively (*p* < 0.05 or 0.001, respectively, Kruskal–Wallis test with Dunn’s multiple comparison) (Figure 5F).

Therefore, the overexpression of Gyc88E in *CNGL* mutant flies suppressed the reduced recovery in response to hypoxia.

### *Pde1c* Mutation Eliminated the Reduced Recovery in Response to Hypoxia in *CNGL* Mutant

Overexpression of Gyc88E, which leads to the upregulation of cGMP, suppresses the w-RNAi induced delay of locomotor recovery (Xiao and Robertson, 2017) and eliminates the reduced recovery in response to hypoxia in *CNGL* mutant. Genes for cGMP-specific PDEs, which regulate intracellular levels of cGMP by hydrolyzing cGMP, have been identified in *Drosophila* (Morton and Hudson, 2002; Day et al., 2005). Therefore, *PDE* mutation provides another approach to investigate the impact of cGMP upregulation (Xiao and Robertson, 2017). One of the *PDE* mutants, Pde1c^KG05572^, could compensate for the *w*-RNAi-induced delay of locomotor recovery from anoxia. Here we examined whether the *Pde1c* mutation could also eliminate the reduced locomotor activity under normoxia and the reduced recovery in response to hypoxia in *CNGL* mutant flies.

**FIGURE 6:**
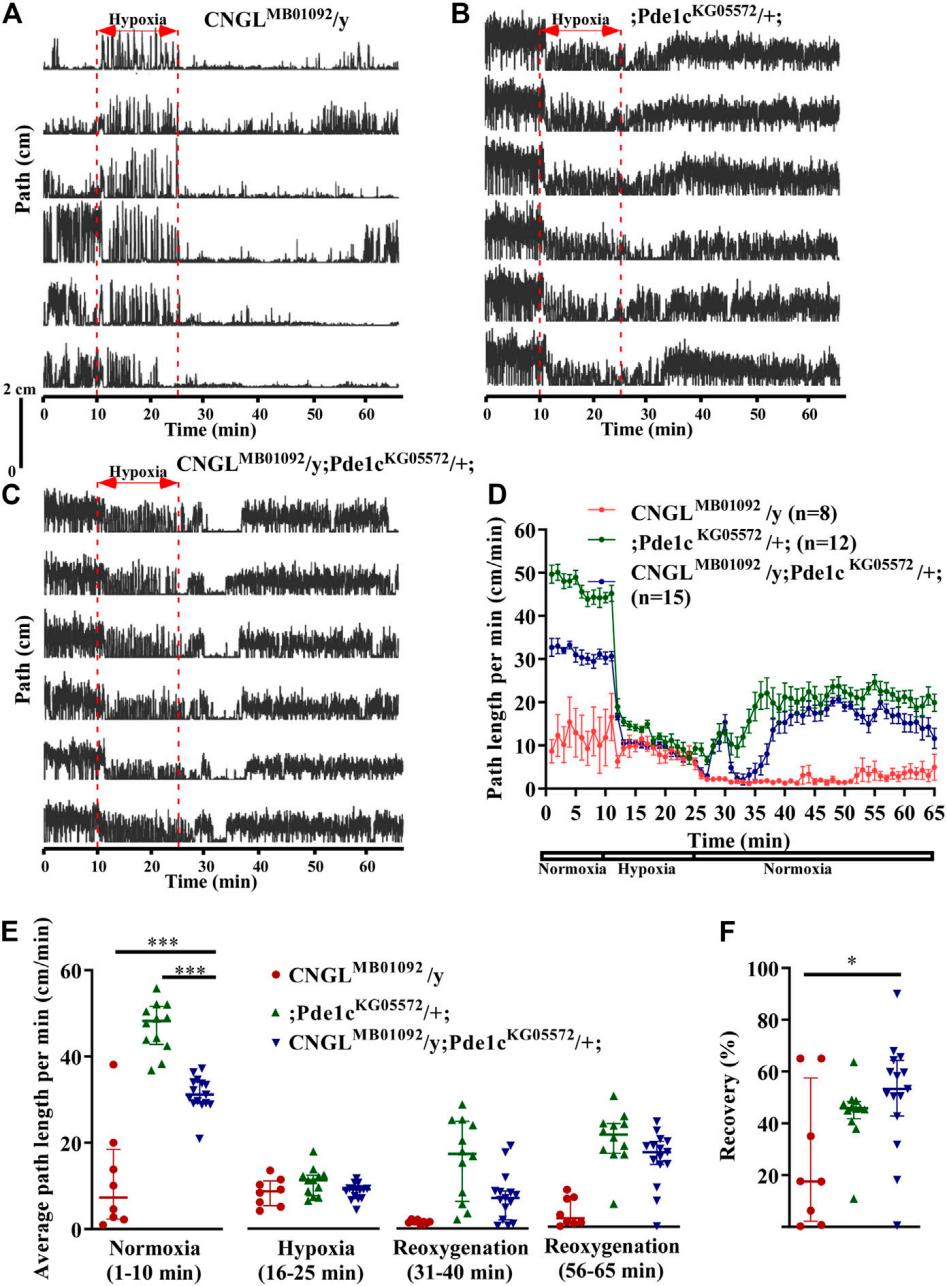
*Pde1c* mutation eliminated both the reduced locomotor activity under normoxia and the reduced recovery in response to hypoxia in *CNGL* mutant. **(A–C)** Locomotor analysis for flies with *Pde1c* mutation and control flies with hypoxia. **(D)** Path length per minute of control flies (CNGL^MB01092^/y, red circles and red line, *n* = 8, and ;*Pde1c* ^KG05572^/+; , blue circles and blue line, *n* = 12) and *CNGL* mutant flies with *Pde1c* mutation (CNGL^MB01092^/y; Pde1c^KG05572^/+; , green circles and green line, *n* = 15) during normoxia, hypoxia and recovery. **(B)** The average path length per minute under normoxia (1–10 min), during hypoxia (16–25 min), and during reoxygenation (31–40 min and 56–65 min) of control flies and *CNGL* mutant flies with *Pde1c* mutation. **(C)** The percent recovery of control flies and *CNGL* mutant flies with *Pde1c* mutation. **(E)** The average path length per minute under normoxia (1–10 min), during hypoxia (16–25 min), and during reoxygenation (31–40 min and 56–65 min) of control flies and *CNGL* mutant flies with *Pde1c* mutation. **(F)** The percent recovery of control and *CNGL* mutant flies with *Pde1c* mutation. Asterisks (* or ***) indicate *p* < 0.05 or 0.001, respectively, by Kruskal–Wallis test with Dunn’s multiple comparison.

Among the three fly genotypes under normoxia, flies CNGL^MB01092^/y; Pde1c^KG05572^/+; displayed better locomotor activity compared with *CNGL* mutant flies (Figures 6A–D). The average path length per minute in CNGL^MB01092^/y; Pde1c^KG05572^ */+*; was 31.2 cm/min (*n* = 15, IQR 29.2-34.0 cm/min). Even though it was a smaller path length per minute than flies; Pde1c^KG05572^/+; (*n* = 12, median 48.2 cm/min, IQR 42.8-51.7 cm/min), it was more than for *CNGL* mutant flies CNGL^MB01092^/y flies (*n* = 8, median 7.3 cm/min, IQR 2.4-18.5 cm/min) (*p* < 0.001 for both comparisons, Kruskal–Wallis test with Dunn’s multiple comparison) (Figure 6E, left panel). These data indicate that Pde1c mutation suppressed the reduced locomotor activity under normoxia in *CNGL* mutant. In addition, O_2_ level changes affected fly locomotor activities in all three fly genotypes (*p* < 0.001 for all three comparisons within same genotypes, Kruskal–Wallis test without post-hoc test) (Figure 6E). Similarly, the path length per minute in CNGL^MB01092^/y flies reached a peak at the beginning of the hypoxia treatment and immediately dropped ∼10 cm/min, which was then followed by a gradual increase (Figures 6A,D).

During recovery, flies CNGL^MB01092^/y; Pde1c^KG05572^/+; walked farther relative to CNGL^MB01092^/y flies (Figures 6A–D). Although the recovery percentage in CNGL^MB01092^/y; Pde1c^KG05572^ */+*; (*n* = 15, median 53.3%, IQR 42.8-64.4%) was comparable with that of ;Pde1c^KG05572^ */+*; flies (*n* = 12, median 46.0%, IQR 45.1-49.0%), the value of CNGL^MB01092^/y; Pde1c^KG05572^ */+*; was significantly higher than that of CNGL^MB01092^/y flies (*n* = 8, median 17.5%, IQR 4.6-43.8%) (*p* > 0.05 or <0.05, respectively, Kruskal–Wallis test with Dunn’s multiple comparison) (Figure 6F). Therefore, the result suggests that the mutation in *Pde1c* eliminated the reduced recovery percentage in response to hypoxia in *CNGL* mutant flies.

## Discussion

CNGL^MB01092^/y mutant w1118 flies had low levels of *CNGL* transcripts and reduced locomotor activity in normoxia compared with control w1118 flies. Reduced locomotor activity in the mutant was a result of more time spent motionless in between episodes of walking and a lower walking speed during episodes. In control flies, hypoxia immediately reduced locomotor activity measured by path length per minute, however there was complete recovery upon the return of air (∼40 min to recover after 15 min of hypoxia). However, CNGL^MB01092^/y flies had an immediate and transient increase in path length per minute at the beginning of hypoxia, followed by a gradual increase during the hypoxia period and poor recovery from hypoxia. *CNGL* knockdown in neurons replicated the properties of the CNGL^MB01092^/y mutant. However, *CNGL* knockdown in glial cells impaired locomotor activity to such an extent that it was not possible to characterize effects of hypoxia and return to air. Finally, we showed that crossing the mutant with UAS-Gyc88E or the *Pde1c* ^KG05572^ mutant, both of which would upregulate cGMP, compensated for *CNGL* downregulation and enabled locomotor recovery from hypoxia in the mutant line.

We show that *CNGL* transcript level is lower in the w1118 CNGL^MB01092^/y mutants than in w1118 controls, however we do not know the effect of this on channel abundance, kinetics or ion currents. Our conclusions therefore relate to the clear and undeniable effects of this genetic mutation on locomotor activity and responses to hypoxia rather than to a defined manipulation of channel function. We propose that CNGL channel activity is reduced in the mutant but this requires confirmation in future experiments. In addition, although the genetic backgrounds of both control and mutant lines were w1118, it remains a possibility that other genetic factors may have influenced the results. However, the effects of the mutation, which reduced levels of *CNGL* transcript, were recapitulated by RNAi targeting *CNGL* in neurons and were compensated by two independent manipulations (Gyc88E and Pde1c) that would increase cGMP levels. Given previous published data on CNG channels, responses to hypoxia in larvae and the role of a *white*/cGMP/PKG signaling pathway in responses of adult flies to anoxia (Dawson-Scully et al., 2010; Vermehren-Schmaedick et al., 2010; Spong et al., 2016; Xiao and Robertson, 2017), we propose that our results were due to changes in CNGL ion channel content and activity.

We found that CNGL^MB01092^/y had reduced locomotor activity under normoxia, suggesting that *CNGL* is necessary for normal motor activity in adult flies. This notion is supported by the structural similarity of CNGL with cyclic nucleotide-gated HCN channels (Xu and Shaw, 2019), which are intimately involved in the central generation of motor patterns in many taxa (Picton et al., 2018). Indeed, it is intriguing that many of the effects of the *CNGL* mutation on fly walking are like the effects of blocking HCN channels on swimming of larval *Xenopus*, including shorter episodes of slower locomotion. In larval *Xenopus*, HCN currents compensate for the hyperpolarizing activity of the Na^+^/K^+^-ATPase linking the locomotor effects to ion homeostasis in the central circuitry (Picton et al., 2018). Alternatively, the increased duration of quiescence with fewer episodes of walking may have been due to a disruption of neuromodulation resulting from the down-regulation of *CNGL*. In larval zebrafish, fictive swimming with an episodic pattern is evoked by the application of N-methyl-d-aspartate (NMDA), which is an activator of the larval zebrafish swimming central pattern generator (McDearmid and Drapeau, 2006; Wiggin et al., 2012). In *C. elegans* episodic locomotion is also observed with spontaneous alternation between active swimming and inactivity, and this transition is promoted by acetylcholine (ACh) signaling (Ghosh and Emmons, 2008). So far, no direct evidence has been found for the involvement of the CNGL channel in the release of ACh, however, CNG channels can modulate glutamatergic synaptic transmission (Zagotta and Siegelbaum, 1996; Bradley et al., 1997; Savchenko et al., 1997).

The CNGL channel is critical for normal locomotor activity in adult flies. CNGL^MB01092^/y flies showed reduced locomotor activity and slower recovery after hypoxia, effects which may both be consequences of the same deficiency. This was comparable with *CNGL* knockdown in neurons, however, a full comparison with *CNGL* knockdown in glia was not possible because of the greatly impaired locomotor activity in these flies. Indeed, RNAi-mediated knockdown of *CNGL* in either neurons or glia had more extreme effects than the mutation, suggesting that the mutant retained more CNGL function. The *CNGL* gene is highly expressed in the nervous tissue, particularly in neuropil regions of the brain (Miyazu et al., 2000), indicating a role in neuronal circuit operation as noted above. However, it is unclear how much CNGL is in glia, which is also critical for neural function and behavior by regulating ion homeostasis and synaptic transmission. It is likely that the CNGL channels in glial cells have different functions from the CNGL channels in neurons, leading to different sensitivities to hypoxia or varied effects of O_2_ level changes on locomotor activities. Indeed, the flies with *CNGL* knockdown in glia were more sensitive to hypoxia to the extent that they convulsed and entered anoxic coma with O_2_ levels that were permissive for locomotor activity in all the other genotypes tested. This is an interesting observation that deserves further research in the context of the role of glia in controlling ion homeostasis and regulating anoxic depolarization in the brain (Armstrong et al., 2011). Nevertheless, compared with Gal4 control flies, the reduced recovery of flies with *CNGL* knockdown in neurons suggests that CNGL channels expressed in neurons regulate hypoxic recovery. To determine the contributions of specific neuronal or glial cell types to locomotor activity or the response to hypoxia, specific driver lines for neurons or glia of the fly CNS could be used to cross with UAS-CNGL-RNAi flies.

The behavioral response to hypoxia in *Drosophila* larvae depends on the nitric oxide (NO)/cyclic GMP (cGMP) signaling pathway (Wingrove and O’farrell, 1999). In swimming *Xenopus laevis* larvae the application of S-nitroso-N-acetylpenicillamine (SNAP) to increase NO levels, results in a reduced episode duration (the time for which an animal swims in response to a brief sensory stimulus) and an increased cycle period (interval between the onset of a burst in one cycle and the onset of the burst in the next cycle) (McLean and Sillar, 2000). On the other hand, treatment with nitric oxide synthase (NOS) inhibitors, such as l-NAME and L-NNA, to reduce NO levels increases the episode duration and decreases the cycle period. The effect of SNAP on the swimming motor pattern is similar to that of sodium azide (NaN_3_) when it is applied to *Xenopus laevis* larvae (Robertson et al., 2010), which is widely used to induce chemical hypoxia in neurons (Varming et al., 1996; Jørgensen et al., 1999; Selvatici et al., 2009) and is reported to generate NO (Smith et al., 1991; Smith and Wilcox, 1994). In adult flies, it is still unclear why an acute increase of locomotor activity was generated at the beginning of hypoxia, especially in the flies with *CNGL* down-regulation or pan-neuronal knockdown. However, it demonstrates that the locomotor system can operate faster. Reduced locomotor activity followed by the acute increase in the fly lines noted above as well as the decreased activity in all other tested fly lines during hypoxia might be due to increased levels of NO, reducing episode duration and thus decreasing the path length per minute. An intriguing possibility is that CNGL potentiates motor patterning and hypoxic activation of other CNG channels can compensate for its knockdown.

The hypoxia escape response in *Drosophila* larvae requires functional CNGA channels but not CNGL channels (Vermehren-Schmaedick et al., 2010). This is consistent with the observation that CNGA channels promote the mobilization of calcium downstream of hypoxia-induced NO signaling but CNGL channels do not (Dijkers and O’farrell, 2009). cGMP modulates the speed of anoxic recovery in adult flies (Xiao and Robertson, 2017) and mediates the escape response to hypoxia in *Drosophila* larvae (Vermehren-Schmaedick et al., 2010). One of the cGMP downstream targets, PKG, is involved in behavioral tolerance to anoxia and hypoxia (Dawson-Scully et al., 2010; Spong et al., 2016). Our results show that the CNGL channel, one member of the CNG ion channel family, which represents downstream targets of cGMP, also regulated the hypoxia response in adult flies. However, whether the hypoxia response in adults also requires the other members of CNG channels such as CNGA, was not investigated in our research. In *Drosophila* larvae, hypoxia activates two heterodimeric atypical sGCs (Gyc-88E/Gyc-89Da and Gyc-88E/Gyc-89Db), which act as O_2_ sensors (Morton, 2011). The sGCs then promote the production of cGMP, which activates CNG channels. *Drosophila* larvae withdraw from food in response to hypoxia (Vermehren-Schmaedick et al., 2010) or remain moving in anoxia for almost 40 min (Callier et al., 2015), whereas adults show complete paralysis within 30 s under anoxia (Callier et al., 2015). To examine the involvement of O_2_ sensors in adult flies, the hypoxia responses in the atypical sGCs mutant flies should be compared with the controls.

We also found that the overexpression of Gyc88E or the mutation of *Pde1c* compensated for the reduced recovery from hypoxia in *CNGL* mutant flies. We did not measure cGMP levels when Gyc88E was overexpressed, however, the overexpression of Gyc88E eliminates the *white* (*w*) gene-RNAi-induced delay of locomotor recovery and the effect is similar to several PDE mutants, especially cGMP-specific PDE mutants (Xiao and Robertson, 2017), suggesting that the Gyc88E overexpression is likely to have increased cGMP levels. Genes for cGMP-specific PDEs, which also regulate intracellular levels of cGMP by hydrolyzing cGMP, have been identified in *Drosophila* (Morton and Hudson, 2002; Day et al., 2005). Therefore, *PDE* mutation is another approach to investigate the impact of cGMP upregulation (Xiao and Robertson, 2017). One of the *PDE* mutants, Pde1c^KG05572^, compensated for the *w*-RNAi-induced delay of locomotor recovery from anoxia. It should be noted that ;UAS-Gyc88E/+; recovered poorly from hypoxia. The reason for this inconsistency between these two approaches might be that the Pde1c mutation was not a UAS-line and therefore, it lost tissue specificity and could not function as specifically as the UAS-Gyc88E fly line, in which Gyc88E was overexpressed only in *CNGL*-positive cells. The reason for this is unclear, however, compared with *CNGL* mutant flies CNGL^MB01092^/y, the remarkable increased recovery of the flies with Gyc88E overexpression or *Pde1c* mutation in *CNGL* mutants suggests that cGMP upregulation eliminated the impaired recovery induced by the down-regulation of *CNGL* in response to hypoxia. cGMP upregulation may have potentiated the activity of remaining CNGL channels, however it is also possible that it compensated for the mutation by activating an alternative downstream pathway.

In summary, our results support the conclusion that the CNGL channel is required for proper locomotor activity and its recovery from hypoxia in *Drosophila* adults. With respect to central pattern generation, the role of cGMP and CNGL channels in episodic motor activity are future research areas that are likely to be particularly fruitful.

## Data Availability

The raw data supporting the conclusion of this article will be made available by the authors, without undue reservation.

## References

[B1] Allweis C. Gibbs M. E. Ng K. T. Hodge R. J. (1984). Effects of Hypoxia on Memory Consolidation: Implications for a Multistage Model of Memory. Behav. Brain Res. 11, 117–121. 10.1016/0166-4328(84)90134-7 6704232

[B2] Armstrong G. A. B. Xiao C. Krill J. L. Seroude L. Dawson-Scully K. Robertson R. M. (2011). Glial Hsp70 Protects K+ Homeostasis in the Drosophila Brain during Repetitive Anoxic Depolarization. PLoS One 6, e28994. 10.1371/journal.pone.0028994 22174942 PMC3236231

[B3] Bellen H. J. Levis R. W. He Y. Carlson J. W. Evans-Holm M. Bae E. (2011). The Drosophila Gene Disruption Project: Progress Using Transposons with Distinctive Site Specificities. Genetics 188, 731–743. 10.1534/genetics.111.126995 21515576 PMC3176542

[B4] Bradley J. Zhang Y. Bakin R. Lester H. A. Ronnett G. V. Zinn K. (1997). Functional Expression of the Heteromeric "olfactory" Cyclic Nucleotide-Gated Channel in the hippocampus: a Potential Effector of Synaptic Plasticity in Brain Neurons. J. Neurosci. 17, 1993–2005. 10.1523/jneurosci.17-06-01993.1997 9045728 PMC6793760

[B58] Brand A. H. Perrimon N. (1993). Targeted Gene Expression as a Means of Altering Cell Fates and Generating Dominant Phenotypes. Development 118, 401–15. 10.1242/dev.118.2.401 8223268

[B5] Callier V. Hand S. C. Campbell J. B. Biddulph T. Harrison J. F. (2015). Developmental Changes in Hypoxic Exposure and Responses to Anoxia in *Drosophila melanogaster* . J. Exp. Biol. 218, 2927–2934. 10.1242/jeb.125849 26206351

[B6] Campbell J. B. Andersen M. K. Overgaard J. Harrison J. F. (2018). Paralytic Hypo-Energetic State Facilitates Anoxia Tolerance Despite Ionic Imbalance in Adult *Drosophila melanogaster* . J. Exp. Biol. 221, jeb177147. 10.1242/jeb.177147 29615525

[B7] Campbell J. B. Overby P. F. Gray A. E. Smith H. C. Harrison J. F. (2019a). Genome-wide Association Analysis of Anoxia Tolerance in *Drosophila melanogaster* . G3: Genes Genomes Genet. 9, 2989–2999. 10.1534/g3.119.400421 PMC6723132 31311780

[B8] Campbell J. B. Werkhoven S. Harrison J. F. (2019b). Metabolomics of Anoxia Tolerance in *Drosophila melanogaster*: Evidence against Substrate Limitation and for Roles of Protective Metabolites and Paralytic Hypometabolism. Am. J. Physiol. Regulat. Integr. Comp. Physiol. 317, R442–R450. 10.1152/ajpregu.00389.2018 31322917

[B9] Chang A. J. Chronis N. Karow D. S. Marletta M. A. Bargmann C. I. (2006). A Distributed Chemosensory Circuit for Oxygen Preference in *C. elegans* . Plos Biol. 4, e274. 10.1371/journal.pbio.0040274 16903785 PMC1540710

[B61] Clemens J. C. Worby C. A. Simonson-Leff N. Muda M. Maehama T. Hemmings B. A. (2000). Use of Double-Stranded RNA Interference in *Drosophila* Cell Lines to Dissect Signal Transduction Pathways. Proc Natl Acad Sci U S A 97, 6499–503. 10.1073/pnas.110149597 10823906 PMC18635

[B10] Cummins T. R. Donnelly D. F. Haddad G. G. (1991). Effect of Metabolic Inhibition on the Excitability of Isolated Hippocampal CA1 Neurons: Developmental Aspects. J. Neurophysiol. 66, 1471–1482. 10.1152/jn.1991.66.5.1471 1662712

[B11] Dawson-Scully K. Bukvic D. Chakaborty-Chatterjee M. Ferreira R. Milton S. L. Sokolowski M. B. (2010). Controlling Anoxic Tolerance in Adult Drosophila *via* the cGMP-PKG Pathway. J. Exp. Biol. 213, 2410–2416. 10.1242/jeb.041319 20581270 PMC2892421

[B12] Day J. P. Dow J. A. T. Houslay M. D. Davies S.-A. (2005). Cyclic Nucleotide Phosphodiesterases in *Drosophila melanogaster* . Biochem. J. 388, 333–342. 10.1042/bj20050057 15673286 PMC1186723

[B13] Dijkers P. F. O'farrell P. H. (2009). Dissection of a Hypoxia-Induced, Nitric Oxide-Mediated Signaling Cascade. MBoC 20, 4083–4090. 10.1091/mbc.e09-05-0362 19625446 PMC2743626

[B14] Dimitroff B. Howe K. Watson A. Campion B. Lee H.-G. Zhao N. (2012). Diet and Energy-Sensing Inputs Affect TorC1-Mediated Axon Misrouting but Not TorC2-Directed Synapse Growth in a *Drosophila* Model of Tuberous Sclerosis. PLOS ONE 7, e30722. 10.1371/journal.pone.0030722 22319582 PMC3272037

[B15] Donohoe P. H. West T. G. Boutilier R. G. (2000). Factors Affecting Membrane Permeability and Ionic Homeostasis in the Cold-Submerged Frog. J. Exp. Biol. 203, 405–414. 10.1242/jeb.203.2.405 10607550

[B60] Duffy J. B. (2002). GAL4 System in *Drosophila*: A Fly Geneticist’s Swiss Army Knife. Genesis 34, 1–15. 10.1002/gene.10150 12324939

[B16] Ghosh R. Emmons S. W. (2008). Episodic Swimming Behavior in the nematodeC. Elegans. J. Exp. Biol. 211, 3703–3711. 10.1242/jeb.023606 19011210

[B17] Gray J. M. Karow D. S. Lu H. Chang A. J. Chang J. S. Ellis R. E. (2004). Oxygen Sensation and Social Feeding Mediated by a *C. elegans* Guanylate Cyclase Homologue. Nature 430, 317–322. 10.1038/nature02714 15220933

[B18] Grima B. Chélot E. Xia R. Rouyer F. (2004). Morning and Evening Peaks of Activity Rely on Different Clock Neurons of the *Drosophila* Brain. Nature 431, 869–873. 10.1038/nature02935 15483616

[B19] Haddad G. G. (2006). Tolerance to Low O2: Lessons from Invertebrate Genetic Models. Exp. Physiol. 91, 277–282. 10.1113/expphysiol.2005.030767 16431936

[B59] Hammond S. M. Bernstein E. Beach D. Hannon G. J. (2000). An RNA-Directed Nuclease Mediates Post-Transcriptional Gene Silencing in *Drosophila* Cells. Nature 404, 293–6. 10.1038/35005107 10749213

[B20] Hoback W. W. Stanley D. W. Higley L. G. Barnhart M. C. (1998). Survival of Immersion and Anoxia by Larval Tiger Beetles, *Cicindela Togata* . Am. Midl. Nat. 140, 27–33. 10.1674/0003-0031(1998)140[0027:soiaab]2.0.co;2

[B21] Hoback W. W. Podrabsky J. E. Higley L. G. Stanley D. W. Hand S. C. (2000). Anoxia Tolerance of Con-Familial Tiger Beetle Larvae Is Associated with Differences in Energy Flow and Anaerobiosis. J. Comp. Physiol. B: Biochem. System. Environ. Physiol. 170, 307–314. 10.1007/s003600000104 10935521

[B22] Jiang C. Haddad G. G. (1994). A Direct Mechanism for Sensing Low Oxygen Levels by central Neurons. Proc. Natl. Acad. Sci. 91, 7198–7201. 10.1073/pnas.91.15.7198 8041769 PMC44366

[B23] Jiang C. Sigworth F. Haddad G. (1994). Oxygen Deprivation Activates an ATP-Inhibitable K+ Channel in Substantia Nigra Neurons. J. Neurosci. 14, 5590–5602. 10.1523/jneurosci.14-09-05590.1994 8083755 PMC6577106

[B24] Jørgensen N. K. Petersen S. F. Damgaard I. Schousboe A. Hoffmann E. K. (1999). Increases in [Ca^2+^]i and Changes in Intracellular pH during Chemical Anoxia in Mouse Neocortical Neurons in Primary Culture. J. Neurosci. Res. 56, 358–370. 10.1002/(SICI)1097-4547(19990515)56:4<358::AID-JNR4>3.0.CO;2-G 10340744

[B25] Kuntz S. Poeck B. Strauss R. (2017). Visual Working Memory Requires Permissive and Instructive NO/cGMP Signaling at Presynapses in the *Drosophila* central Brain. Curr. Biol. 27, 613–623. 10.1016/j.cub.2016.12.056 28216314

[B26] López-Barneo J. (1996). Oxygen-sensing by Ion Channels and the Regulation of Cellular Functions. Trends Neurosci. 19, 435–440. 10.1016/0166-2236(96)10050-3 8888521

[B27] López-Barneo J. López-López J. R. Ureña J. Gonzalez C. (1988). Chemotransduction in the Carotid Body: K + Current Modulated by PO_2_ in Type I Chemoreceptor Cells. Science 241, 580–582. 10.1126/science.2456613 2456613

[B28] Luo L. Liao Y. J. Jan L. Y. Jan Y. N. (1994). Distinct Morphogenetic Functions of Similar Small GTPases: *Drosophila* Drac1 Is Involved in Axonal Outgrowth and Myoblast Fusion. Genes Dev. 8, 1787–1802. 10.1101/gad.8.15.1787 7958857

[B29] McDearmid J. R. Drapeau P. (2006). Rhythmic Motor Activity Evoked by NMDA in the Spinal Zebrafish Larva. J. Neurophysiol. 95, 401–417. 10.1152/jn.00844.2005 16207779

[B30] McLean D. L. Sillar K. T. (2000). The Distribution of NADPH-Diaphorase-Labelled Interneurons and the Role of Nitric Oxide in the Swimming System of *Xenopus laevis* Larvae. J. Exp. Biol. 203, 705–713. 10.1242/jeb.203.4.705 10648212

[B31] Metaxakis A. Oehler S. Klinakis A. Savakis C. (2005). Minos as a Genetic and Genomic Tool in *Drosophila melanogaster* . Genetics 171, 571–581. 10.1534/genetics.105.041848 15972463 PMC1456772

[B32] Miyazu M. Tanimura T. Sokabe M. (2000). Molecular Cloning and Characterization of a Putative Cyclic Nucleotide-Gated Channel from *Drosophila melanogaster* . Insect Mol. Biol. 9, 283–292. 10.1046/j.1365-2583.2000.00186.x 10886412

[B33] Morton D. B. Hudson M. L. (2002). “Cyclic GMP Regulation and Function in Insects,” in Advances in Insect Physiology (Cambridge, MA: Academic Press), 1–54. 10.1016/s0065-2806(02)29001-3

[B34] Morton D. B. (2004). Atypical Soluble Guanylyl Cyclases in *Drosophila* Can Function as Molecular Oxygen Sensors. J. Biol. Chem. 279, 50651–50653. 10.1074/jbc.c400461200 15485853

[B35] Morton D. B. (2011). Behavioral Responses to Hypoxia and Hyperoxia in Drosophila Larvae. Fly 5, 119–125. 10.4161/fly.5.2.14284 21150317 PMC3127060

[B36] Nagell B. Landahl C.-C. (1978). Resistance to Anoxia of *Chironomus Plumosus* and *Chironomus Anthracinus* (Diptera) Larvae. Ecography 1, 333–336. 10.1111/j.1600-0587.1978.tb00968.x

[B37] Picton L. D. Sillar K. T. Zhang H.-Y. (2018). Control of *Xenopus* Tadpole Locomotion *via* Selective Expression of Ih in Excitatory Interneurons. Curr. Biol. 28, 3911–3923. 10.1016/j.cub.2018.10.048 30503615 PMC6303192

[B38] Robertson R. M. Björnfors E. R. Sillar K. T. (2010). Long-lasting Effects of Chemical Hypoxia on Spinal Cord Function in Tadpoles. Neuroreport 21, 943–947. 10.1097/wnr.0b013e32833e332d 20697300

[B39] Robertson R. M. Dawson-Scully K. D. Andrew R. D. (2020). Neural Shutdown under Stress: an Evolutionary Perspective on Spreading Depolarization. J. Neurophysiol. 123, 885–895. 10.1152/jn.00724.2019 32023142 PMC7099469

[B40] Savchenko A. Barnes S. Kramer R. H. (1997). Cyclic-nucleotide-gated Channels Mediate Synaptic Feedback by Nitric Oxide. Nature 390, 694–698. 10.1038/37803 9414163 PMC3858101

[B41] Selvatici R. Previati M. Marino S. Marani L. Falzarano S. Lanzoni I. (2009). Sodium Azide Induced Neuronal Damage *In Vitro*: Evidence for Non-apoptotic Cell Death. Neurochem. Res. 34, 909–916. 10.1007/s11064-008-9852-0 18841470

[B42] Sepp K. J. Schulte J. Auld V. J. (2001). Peripheral Glia Direct Axon Guidance across the CNS/PNS Transition Zone. Develop. Biol. 238, 47–63. 10.1006/dbio.2001.0411 11783993

[B43] Smith R. P. Wilcox D. E. (1994). Toxicology of Selected Nitric Oxide-Donating Xenobiotics, with Particular Reference to Azide. Crit. Rev. Toxicol. 24, 355–377. 10.3109/10408449409017923 7857522

[B44] Smith R. P. Louis C. A. Kruszyna R. Kruszyna H. (1991). Acute Neurotoxicity of Sodium Azide and Nitric Oxide. Toxicol. Sci. 17, 120–127. 10.1093/toxsci/17.1.120 1916070

[B45] Spong K. E. Rodríguez E. C. Robertson R. M. (2016). Spreading Depolarization in the Brain of Drosophila Is Induced by Inhibition of the Na+/K+-ATPase and Mitigated by a Decrease in Activity of Protein Kinase G. J. Neurophysiol. 116, 1152–1160. 10.1152/jn.00353.2016 27358319 PMC5013169

[B46] Van Voorhies W. A. (2009). Metabolic Function in *Drosophila melanogaster* in Response to Hypoxia and Pure Oxygen. J. Exp. Biol. 212, 3132–3141. 10.1242/jeb.031179 19749106 PMC2742449

[B47] Varming T. Drejer J. Frandsen A. Schousboe A. (1996). Characterization of a Chemical Anoxia Model in Cerebellar Granule Neurons Using Sodium Azide: Protection by Nifedipine and MK-801. J. Neurosci. Res. 44, 40–46. 10.1002/(sici)1097-4547(19960401)44:1<40::aid-jnr5>3.0.co;2-i 8926628

[B48] Vermehren-Schmaedick A. Ainsley J. A. Johnson W. A. Davies S.-A. Morton D. B. (2010). Behavioral Responses to Hypoxia in *Drosophila* Larvae Are Mediated by Atypical Soluble Guanylyl Cyclases. Genetics 186, 183–196. 10.1534/genetics.110.118166 20592263 PMC2940286

[B49] Wiggin T. D. Anderson T. M. Eian J. Peck J. H. Masino M. A. (2012). Episodic Swimming in the Larval Zebrafish Is Generated by a Spatially Distributed Spinal Network with Modular Functional Organization. J. Neurophysiol. 108, 925–934. 10.1152/jn.00233.2012 22572943 PMC3424096

[B50] Wingrove J. A. O'farrell P. H. (1999). Nitric Oxide Contributes to Behavioral, Cellular, and Developmental Responses to Low Oxygen in *Drosophila* . Cell 98, 105–114. 10.1016/s0092-8674(00)80610-8 10412985 PMC2754235

[B51] Wu B. S. Lee J. K. Thompson K. M. Walker V. K. Moyes C. D. Robertson R. M. (2002). Anoxia Induces Thermotolerance in the Locust Flight System. J. Exp. Biol. 205, 815–827. 10.1242/jeb.205.6.815 11914390

[B52] Xiao C. Robertson R. M. (2015). Locomotion Induced by Spatial Restriction in Adult *Drosophila* . PLoS ONE 10, e0135825. 10.1371/journal.pone.0135825 26351842 PMC4564261

[B53] Xiao C. Robertson R. M. (2017). White - cGMP Interaction Promotes Fast Locomotor Recovery from Anoxia in Adult *Drosophila* . PLOS ONE 12, e0168361. 10.1371/journal.pone.0168361 28060942 PMC5218474

[B54] Xiao C. Qiu S. Robertson R. M. (2018). Persistent One-Way Walking in a Circular arena in *Drosophila melanogaster* Canton-S Strain. Behav. Genet. 48, 80–93. 10.1007/s10519-017-9881-z 29098495

[B55] Xu M. Shaw K. L. (2019). The Genetics of Mating Song Evolution Underlying Rapid Speciation: Linking Quantitative Variation to Candidate Genes for Behavioral Isolation. Genetics 211, 1089–1104. 10.1534/genetics.118.301706 30647070 PMC6404256

[B56] Zagotta W. N. Siegelbaum S. A. (1996). Structure and Function of Cyclic Nucleotide-Gated Channels. Annu. Rev. Neurosci. 19, 235–263. 10.1146/annurev.ne.19.030196.001315 8833443

[B57] Zimmer M. Gray J. M. Pokala N. Chang A. J. Karow D. S. Marletta M. A. (2009). Neurons Detect Increases and Decreases in Oxygen Levels Using Distinct Guanylate Cyclases. Neuron 61, 865–879. 10.1016/j.neuron.2009.02.013 19323996 PMC2760494

